# Supplementary material for: Integrated analysis of the aqueous humor microbiome and lens capsule transcriptome in high myopia cataract: a pilot study
Source: Front Med (Lausanne). 2026 Jun 16;13:1845205. doi: 10.3389/fmed.2026.1845205 (PMC13314463; doi:10.3389/fmed.2026.1845205)
Supplement: Supplementary file 3 [file Data_Sheet_1.zip › 6.PICRUSt2/Meta_Statistic/C372089/Wilcoxon/COG_abundance.Wilcoxon.diff.top10.boxplot.pdf]

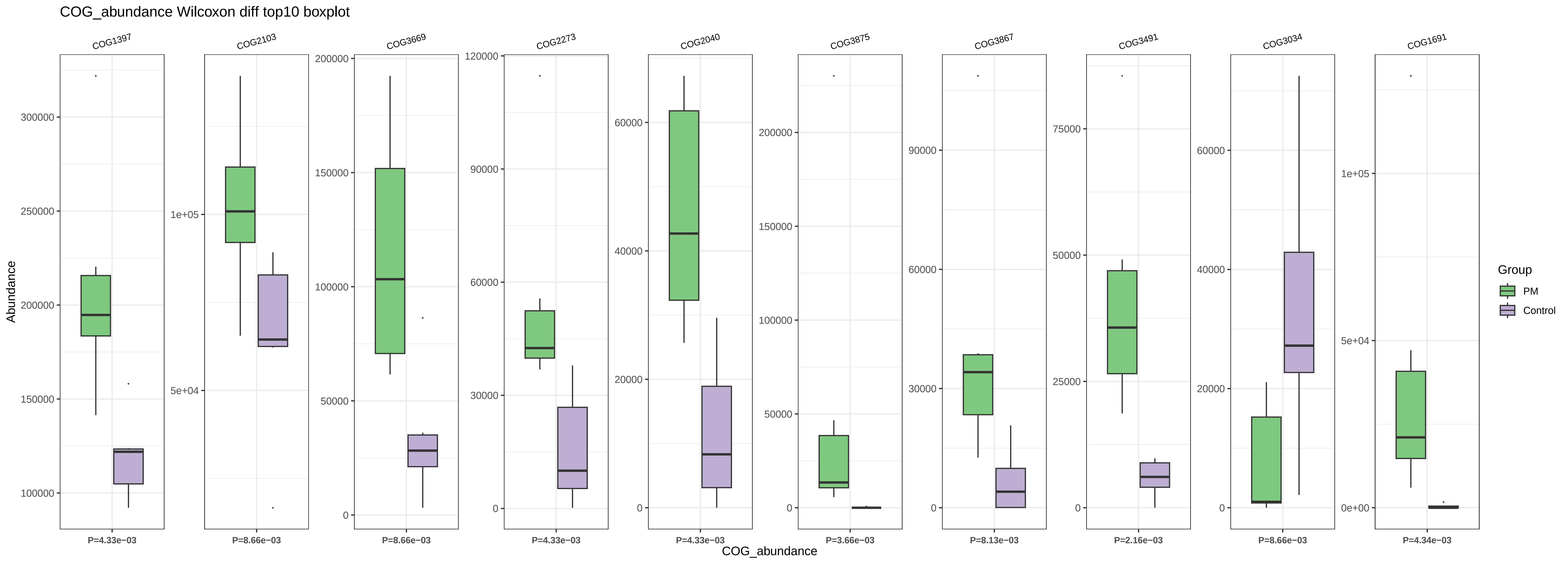
COG_abundance Wilcoxon diff top10 boxplot
Abundance
COG_abundance
COG1397
COG2103
COG3669
COG2273
COG2040
COG3875
COG3867
COG3491
COG3034
COG1691
P=4.33e−03
P=8.66e−03
P=8.66e−03
P=4.33e−03
P=4.33e−03
P=3.66e−03
P=8.13e−03
P=2.16e−03
P=8.66e−03
P=4.34e−03
Group
PM
Control
